# Cellular Immunotherapy in Mice Prevents Maternal Hypertension and Restores Anti-Inflammatory Cytokine Balance in Maternal and Fetal Tissues

**DOI:** 10.3390/ijms241713594

**Published:** 2023-09-02

**Authors:** Gabrielle Gray, Douglas G. Scroggins, Katlin T. Wilson, Sabrina M. Scroggins

**Affiliations:** 1 Carver College of Medicine, University of Iowa, Iowa City, IA 52242, USA; 2 School of Medicine, Department of Biomedical Sciences, University of Minnesota Duluth, Duluth, MN 55812, USA

**Keywords:** cellular therapy, preeclampsia, cytokines, inflammation, regulatory dendritic cells

## Abstract

Preeclampsia is the leading cause of maternal–fetal morbidity worldwide. The concept that persistent feto-placental intolerance is important in the pathogenesis of preeclampsia (PreE) has been demonstrated by our lab and others. Arginine vasopressin (AVP) infusion during pregnancy induces cardiovascular, renal, and T helper (T_H_) cell alterations in mice consistent with human PreE. In addition to their conventional immuno-stimulatory role, dendritic cells (DCs) also play a vital role in immune tolerance. In contrast to conventional DCs, regulatory DCs (DCregs) express low levels of co-stimulatory markers, produce anti-inflammatory cytokines, induce T regulatory (Treg) cells, and promote tolerance. In mice, DCregs prevent pro-inflammatory responses and induce antigen-specific tolerance. Given these known functions of DCregs, we hypothesize that DCregs will prevent the development of AVP-induced PreE in mice. *C57BL/6J* females were infused with AVP (24 ng/h) or saline throughout gestation via an osmotic minipump. Bone-marrow-derived DCregs were injected into AVP-infused dams at the time of the pump implantation or on gestational day (GD) 7. The blood pressure of the mice was taken throughout their pregnancy. The maternal urine proteins and T_H_-associated cytokines in maternal and fetal tissues were measured on GD 18. The treatment with DCregs effectively prevented the elevation of maternal blood pressure, proteinuria, and fetal growth restriction that were observed in AVP-infused dams. Furthermore, we noted a reduction in the pro-inflammatory T_H_-associated cytokines IFNγ and IL-17, while anti-inflammatory cytokines IL-4, IL-10, and TGFβ showed an increase following DCreg treatment. These outcomes provide strong evidence supporting the potential of DCregs as a valuable therapeutic approach in addressing PreE.

## 1. Introduction

Preeclampsia (PreE), a hypertensive disorder in pregnancy, affects 5–7% of all pregnancies in the United States, equating to 400,000 pregnancies annually. It is a leading cause of maternal–fetal mortality worldwide, resulting in approximately 76,000 maternal and 500,000 neonatal deaths each year. PreE causes both immediate and long-term medical complications for the fetus and mother [1,2,3]. Mothers affected by PreE and their children are at an increased risk of developing cardiovascular and metabolic diseases as well as cognitive impairments later in life [4,5,6,7,8,9,10,11,12,13,14,15,16,17,18]. Currently, the only curative option is the often-preterm delivery of the fetus, which contributes to considerable fetal morbidity and mortality [19], underscoring the importance of developing new modalities to prevent and treat PreE.

The pathogenesis of PreE involves multiple bodily systems, including placental, vascular, renal, and immune dysfunction. Because PreE is a disease resulting from these multiple pathways, successful interventions to prevent and treat PreE must originate from the upstream initiation and regulation of these multiple pathways. Previous work demonstrating that T helper (T_H_) cell dysregulation contributes to poor vascular, renal, and immune function in PreE [20,21,22,23,24,25] supports the concept that immune dysfunction, particularly T_H_ cell dysregulation, may play an etiological role in PreE. The modulation of T cells may be an effective treatment strategy for PreE. Dendritic cells (DCs) are antigen-presenting cells that play a pivotal role in T cell activation and regulation [26,27,28]. In addition to their conventional immuno-stimulatory role in adaptive immunity, DCs also play a pivotal role in immune homeostasis and tolerance through their direct interactions with T cells and cytokine production [26]. In contrast to conventional DCs, regulatory DCs (DCregs) are defined by their ability to induce tolerance. The mechanisms of DCreg function include induction of Treg, T cell anergy, production of soluble factors, and receptor-mediated inhibition [29,30,31,32,33,34,35,36,37]. In animal models, DCregs prevent lethal systemic inflammatory responses, inflammatory bowel disease, allergic airway disease, solid organ allograft rejection, arthritis, and lethal graft vs. host disease [38,39,40,41,42,43,44] and prove an effective therapy for the treatment of allergic airway and autoimmune diseases [45]. Like PreE, immune dysfunction is pathological in these diseases. Therefore, the overall objective of this study was to determine if the administration of DCregs would prevent hypertension and restore immune balance in PreE. To this end, a pregnancy-specific vasopressin (AVP) -induced mouse model of PreE was utilized to assess the hallmark features of PreE: elevated blood pressure, proteinuria, fetal growth restriction, and maternal and fetal tissue cytokine profiles on gestational day 18 (GD18).

## 2. Results

### 2.1. DCreg Administration Prevents Maternal and Fetal PreE-Associated Morbidities

**Figure 1:**
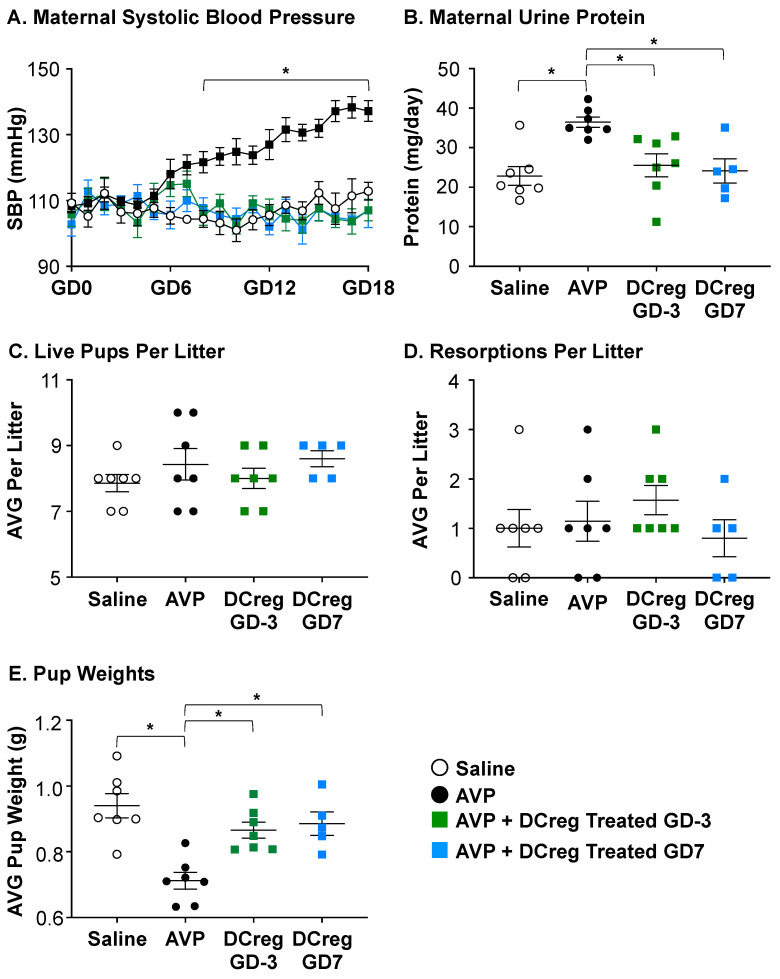
DCreg administration prevents increases in blood pressure and proteinuria in vasopressin-induced preeclampsia. (**A**) Systolic blood pressure throughout gestation in vasopressin-induced preeclampsia. * = AVP was significantly higher than all other groups; (**B**) maternal 24 h urine protein concentrations on GD18; (**C**) average number of live pups per litter; (**D**) average number of resorbed pups per litter; (**E**) average weight of pups in each litter at the time of delivery. AVP = vasopressin; GD = gestational day; N = 5–7 per group. Data are ± SEM. * *p* < 0.05.

Blood pressure was taken throughout gestation via a tail cuff on saline- or AVP-infused dams. As previously published in rodent models [25,46,47,48], AVP infusion during pregnancy resulted in significantly higher systolic blood pressure compared to saline dams beginning at GD8 that persisted throughout gestation (Figure 1A). Urine protein concentration was increased after 24 h (Figure 1B), and lower pup weights were observed (Figure 1E) with no remarkable impact on the size of litters (Figure 1C) or resorptions per litter (Figure 1D). The administration of DCregs at the time of the minipump implantation (GD3) or on GD7 prevented AVP-induced increases in systolic blood pressure beginning on GD8 and persisted throughout gestation (Figure 1A), urine protein (Figure 1B), and growth restriction (Figure 1E). DCreg administration did not alter the number of pups (Figure 1C) or resorptions (Figure 1D) per litter. Collectively, these data support DCreg administration in the prevention of AVP-induced increases in blood pressure, proteinuria, and growth restriction.

### 2.2. DCreg Treatment Prevents Elevated Maternal and Fetal Inflammatory Cytokines

**Figure 2:**
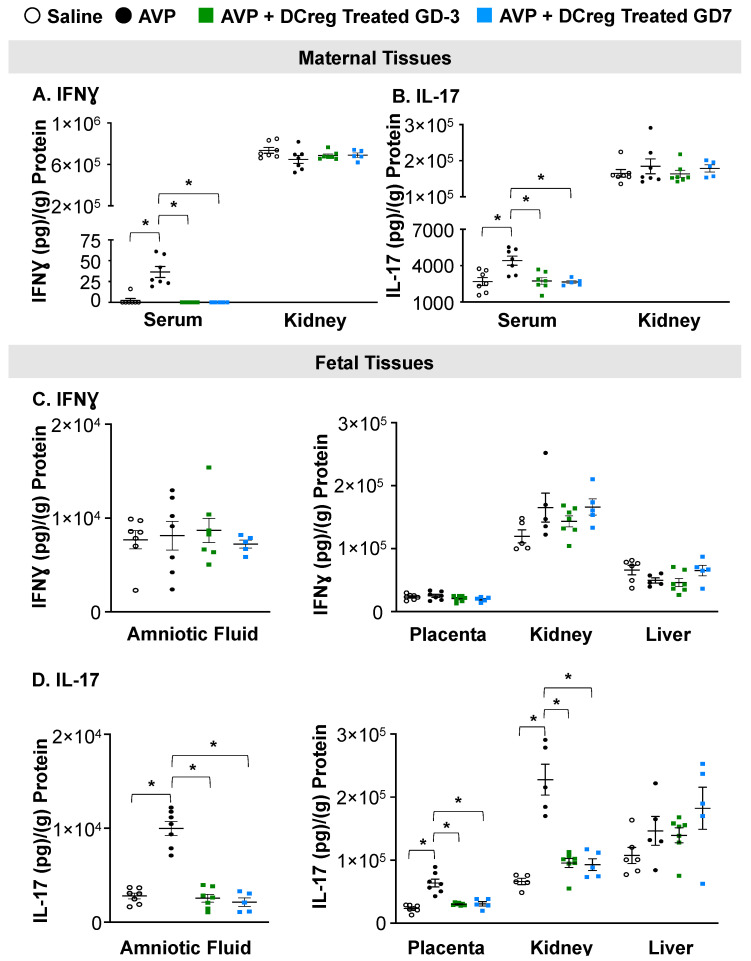
Preeclampsia-induced inflammation in maternal and fetal tissues is ameliorated by maternal DCreg administration. (**A**) IFNƔ and (**B**) IL-17 concentrations in maternal serum and kidney. Concentrations of (**C**) IFNƔ and (**D**) IL-17 in amniotic fluid, placenta, fetal kidney, and fetal liver. AVP = vasopressin; GD = gestational day. N = 5–7 per group. Data are ± SEM. * *p* < 0.05.

Consistent with previous studies, IFNƔ (Figure 2A) and IL-17 (Figure 2B) were increased in the serum of AVP-infused dams compared to saline. DCreg administration significantly reduced IFNƔ and IL-17 in the maternal serum by both GD3 and GD7, with no significant changes in maternal kidney IFNƔ or IL-17 concentrations (Figure 2A and Figure 2B, respectively). The administration of DCreg on either GD3 or GD7 returned the maternal serum IFNƔ (Figure 2A) and IL-17 (Figure 2B) to concentrations comparable to the saline-infused dams. The AVP infusion during gestation did not significantly alter IFNƔ concentrations in the amniotic fluid, placenta, fetal kidney, or fetal liver; and the DCreg administration did not alter IFNƔ concentrations in these fetal tissues (Figure 2C). The maternal AVP-infusion resulted in increased IL-17 in the amniotic fluid, placenta, and fetal kidney (Figure 2D). The maternal DCreg treatment prevented increases in the concentration of IL-17 in the amniotic fluid, placenta, and fetal kidney. The fetal liver IL-17 concentrations were not significantly impacted by maternal AVP-infusion or DCreg administration (Figure 2D).

### 2.3. Treatment with DCreg Restores Maternal and Fetal Tissue Anti-Inflammatory Cytokines

**Figure 3:**
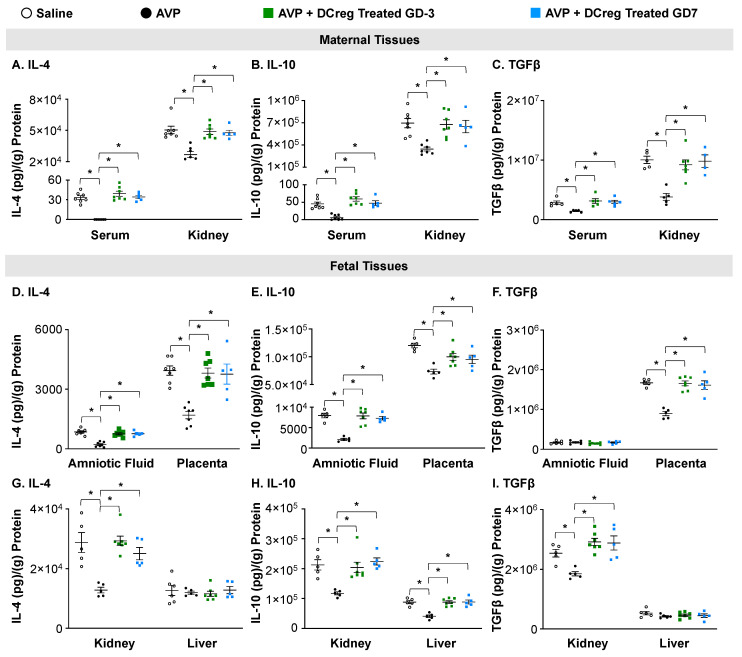
Administration of DCreg upregulates production of anti-inflammatory cytokines in maternal and fetal tissues from preeclampsia-affected pregnancies. Concentrations of (**A**) IL-4, (**B**) IL-10, and (**C**) TGFβ in maternal serum and kidney. Amniotic fluid and placenta concentrations of (**D**) IL-4, (**E**) IL-10, and (**F**) TGFβ. Concentrations of (**G**) IL-4, (**H**) IL-10, and (**I**) TGFβ in fetal kidney and liver at GD18. AVP = vasopressin; GD = gestational day; N = 5–7 per group. Data are ± SEM. * *p* < 0.05.

The AVP-infusion during gestation resulted in decreased anti-inflammatory cytokines IL-4, IL-10, and TGFβ in the maternal serum and kidney. The DCreg treatment prior to mating (GD3) or on GD7 significantly increased IL-4, IL-10, and TGFβ within these tissues (Figure 3A–C). The amniotic fluid, placenta, and fetal kidney IL-4 and IL-10 concentrations were significantly decreased in the offspring from AVP-infused dams (Figure 3D,E,G,H), while TGFβ was significantly decreased in the placenta and fetal kidney (Figure 3F,I). The treatment of AVP-infused dams on GD3 or GD7 prevented an AVP-induced suppression of IL-4, IL-10, and TGFβ in the amniotic fluid, placenta, and fetal kidney (Figure 3D–I). In the fetal liver, only IL-10 was significantly reduced due to AVP-infusion, and DCreg treatment at either timepoint restored the IL-10 levels (Figure 3H).

## 3. Discussion

PreE is the leading cause of maternal–fetal morbidity in both developed and developing countries. Currently, there is a lack of effective interventions to prevent and treat PreE. Immune dysfunction and enhanced T cell-mediated inflammation are key to the pathogenesis of PreE. Therefore, the objective of the current study was to determine if the modulation of immune responses via cellular immunotherapy mitigates the key aspects of PreE.

Although PreE begins very early in pregnancy, one of the first symptoms of PreE in humans and mouse models is elevated blood pressure. Later in gestation, proteinuria and fetal growth restriction are associated with PreE. Indeed, our AVP-induced mouse model demonstrated increased blood pressure, proteinuria, and fetal growth restriction. The administration of DCreg prevented the PreE-induced changes in the vascular, renal, and fetal outcomes. A likely mechanism of DCreg alleviation of PreE is the regulation of the inflammatory T cell responses in PreE.

In instances of PreE, there is an increase in T_H_1 cells producing IFNγ and T_H_17 cells producing IL-17, along with a decrease in T_H_2-related cytokines such as IL-4 and IL-10 [18,21,41,42,43]. This creates an environment abundant in both cells and cytokines that encourage an inflammatory response. While the precise mechanisms responsible for initiating PreE are complex and not fully understood, it is clear that an atypical T_H_ cell response to pregnancy has a significant impact [16]. Indeed, we have observed elevated levels of IFNγ and IL-17, alongside reduced levels of IL-4, IL-10, and TGFβ, in both maternal and fetal tissues from pregnancies affected by PreE, as well as reduced proportions of Treg cells in PreE [25]. These alterations in T_H_-associated cytokines were prevented through the treatment of dams before and early in pregnancy with DCregs.

Dendritic cells (DCs) are widely acknowledged as the primary antigen-presenting cells that contribute to adaptive immunity [25,26]. Beyond their conventional role in immune stimulation, DCs also have a crucial role in maintaining immune balance and tolerance through their interactions with T cells and cytokine secretion [24]. While conventional DCs have their immuno-stimulatory functions, DCregs induce T cell tolerance both in vitro and in vivo. This tolerance-inducing process can happen through direct or indirect mechanisms [35,36,37]. DCregs exhibit a distinctive ratio of co-stimulatory to inhibitory surface molecules. Alongside their minimal expression of co-stimulatory molecules, DCregs also generate anti-inflammatory cytokines [29,30,31,32,33,34,35,36,37].

Various identifying features and functional attributes have been associated with DC populations possessing regulatory capabilities. These include the expression of PD-L1 and PD-L2 [27,29], as well as the production of IL-10 and TGF-β [26,32,33,34,35,40], and indoleamine 2,3 dioxygenase (IDO) [26,43]. DCregs that produce IL-10 and IDO facilitate the differentiation of Tregs. Previous research has strongly indicated the involvement of DCregs in fostering tolerance towards alloantigens [33,42]. In our study, DCreg were able to prevent PreE-induced T_H_ cell-associated dysregulation. A study by Zhang et al. demonstrated that the PD-1/PD-L1 pathway contributes to the imbalance of Treg and T_H_17 cells in PreE [49]. In a previous study, we showed reduced expression of PD-L1 on DCs from PreE-affected dams [25]. In the current study, one potential mechanism for the prevention of PreE-induced inflammation may be via alteration of the PD-1/PD-L1 pathway. Additional studies are ongoing to investigate the role of the PD-1/PD-L1 and other known regulatory pathways in DCreg-mediated PreE prevention.

## 4. Materials and Methods

### 4.1. Generation of DCreg

Eight days before timed mating, syngeneic DCreg cultures were started, as previously described by our group and others [43,50]. Briefly, bone marrow cells from female *C57BL/6J* mice were cultured at 1 × 10^5^ with human TGF-β1, murine GM-CSF, and IL-10 (Peprotech, Cranbury, NJ; 20 ng/mL each). To terminally mature DCregs before intravenous adoptive transfer, LPS (Sigma-Aldrich, St. Louis, MO, USA; 1 μg/mL) was added to DCreg cultures on day 6 of culture. On day 8 of culture, DCreg were isolated from flasks via gentle pipetting and washed with sterile 0.9% sodium chloride (Dechra, Overland Park, KS, USA). Cells were resuspended in 0.9% sodium chloride, and 5 × 10^6^ DCregs were administered intravenously per mouse.

### 4.2. Induction of Preeclampsia

Because PreE is a maternal disease that only occurs in females, only females were used to induce preeclampsia in mice. To ensure accurate gestational calculations, all mating in the current study was for a single over-night period (GD0) using wild-type male *C57BL/6J* mice. Three days prior to mating (GD3), 12-week-old virgin female *C57BL/6J* mice were implanted with subcutaneous osmotic mini-pumps (Alzet, Cupertino, CA, USA) to deliver 24 ng/h AVP (Tocris, BioTechne, Minneapolis, MN, USA) or 0.9% sodium chloride (saline; Vetivex, Dechra Veterinary Products, Leawood, KS, USA) [25,47]. For DCreg-treated groups, DCregs were intravenously administered at the time of pump implantation (GD3) or on GD7. The four experimental groups of dams were: (1) saline (control), (2) AVP-infused (preeclamptic), (3) AVP-infused + GD3 DCreg-treated, and (4) AVP-infused + GD7 DCreg-treated.

On GD18, the dams were euthanized and underwent maternal and fetal necropsy. Litter sizes and pup weights were recorded at the time of maternal euthanasia. Each pregnancy was designated N = 1. Fetal tissues from a single pregnancy were pooled from 5 separate fetuses for an N = 1. Data are pooled from multiple independent experiments. Maternal tissues (serum and kidney) and fetal tissues (amniotic fluid, placenta, kidney, and liver) had N ≥ 5 per group from a minimum of two independent experiments. All samples were stored at −80 °C for subsequent protein extraction, quantification, and cytokine analysis.

### 4.3. Whole Tissue Extraction of Protein and Total Protein Analysis

Whole maternal and fetal tissues were homogenized in buffer containing 5 M NaCl, 1 M Tris, 0.5 M EDTA, NP-40, a protease inhibitor (Roche, Basel, Switzerland), and a phosphatase inhibitor (Roche, Basel, Switzerland). Total protein was quantified using a bicinchoninic acid (BCA) protein assay kit (Thermo Fisher Scientific, Waltham, MA, USA) per the manufacturer’s instructions. ELISAs were used to quantify the pro-inflammatory IFNƔ and IL-17 and anti-inflammatory IL-4, IL-10, and TGFβ of all samples in duplicate (Invitrogen, Thermo Fisher Scientific, Waltham, MA, USA). Cytokine concentrations were normalized to total protein (grams) and are represented as picograms/gram of total protein (pg/g).

### 4.4. Blood Pressure and Proteinuria

Systolic blood pressure (SBP) was obtained as we previously described using the clinically validated CODA Noninvasive Blood Pressure System (Kent Scientific Corp, Torrington, CT, USA) [47,51,52]. Mice were acclimated to the CODA system for 21 days prior to pump implantation. Measurements throughout the study were collected at the same time of day, seven days/week. A total of 60 cycles were collected daily. The first 30 cycles each day were considered daily acclimation and discarded. The last 30 cycles each day were used to calculate the mean daily measurements for each mouse. The last 5 days of recording during the initial 21-day acclimation period were used to calculate baseline blood pressure prior to pump implantation. Experimental blood pressure data were collected throughout GD16. On GD16, dams were placed into metabolic cages for 24 h urine collection. Total protein concentrations of the 24 h GD18 urine were determined using a bicinchoninic acid (BCA) protein assay kit (Thermo Fisher Scientific, Waltham, MA, USA) per the manufacturer’s instructions. Urine protein is reported as mg protein 24 h.

### 4.5. Statistical Analysis

For continuous variables, a two-tailed Student’s *t* test with unequal variance was utilized or a one-way or two-way ANOVA multiple comparisons test where appropriate (GraphPad, Prism 10, La Jolla, CA, USA). Test for outliers were performed in GraphPad and via Tukey’s box-plot method (IQR method). No outliers were identified in this study. Data are expressed as ± standard error of the mean (SEM). The minimal level of confidence designated statistically significant was α = 0.05 or as determined by Bonferroni correction for multiple comparisons ANOVA.

## 5. Conclusions

Our study aimed to determine if DCreg cellular immunotherapy will prevent the cardiovascular, renal, fetal, and immunological alterations associated with PreE. We determined that DCreg treatment prior to pregnancy or early in pregnancy was able to reduce maternal blood pressure, decrease urine protein concentrations, and restore the balance between pro- and anti- inflammatory T_H_ cytokines. Furthermore, the treatment with DCreg did not increase fetal resorption rates or have any observable differences in fetal outcomes by GD18. Thus, this study establishes that DCreg immunotherapy may be a potent therapeutic intervention for PreE.

## Data Availability

The data presented in this study are available upon request from the corresponding author.

## References

[1] Villar J., Carroli G., Wojdyla D., Abalos E., Giordano D., Ba’aqeel H., Farnot U., Bergsjo P., Bakketeig L., Lumbiganon P. (2006). Preeclampsia, gestational hypertension and intrauterine growth restriction, related or independent conditions?. Am. J. Obstet. Gynecol..

[2] (2020). Gestational Hypertension and Preeclampsia: ACOG Practice Bulletin, Number 222. Obs. Gynecol..

[3] Butwick A.J., Druzin M.L., Shaw G.M., Guo N. (2020). Evaluation of US State-Level Variation in Hypertensive Disorders of Pregnancy. JAMA Netw. Open..

[4] Kajantie E., Eriksson J.G., Osmond C., Thornburg K., Barker D.J. (2009). Pre-eclampsia is associated with increased risk of stroke in the adult offspring: The Helsinki birth cohort study. Stroke A J. Cereb. Circ..

[5] Lykke J.A., Langhoff-Roos J., Sibai B.M., Funai E.F., Triche E.W., Paidas M.J. (2009). Hypertensive pregnancy disorders and subsequent cardiovascular morbidity and type 2 diabetes mellitus in the mother. Hypertension.

[6] Wu C.S., Nohr E.A., Bech B.H., Vestergaard M., Catov J.M., Olsen J. (2009). Health of children born to mothers who had preeclampsia: A population-based cohort study. Am. J. Obstet. Gynecol..

[7] Magnussen E.B., Vatten L.J., Smith G.D., Romundstad P.R. (2009). Hypertensive disorders in pregnancy and subsequently measured cardiovascular risk factors. Obs. Gynecol..

[8] Kuklina E.V., Ayala C., Callaghan W.M. (2009). Hypertensive disorders and severe obstetric morbidity in the United States. Obs. Gynecol..

[9] Baecke M., Spaanderman M.E., van der Werf S.P. (2009). Cognitive function after pre-eclampsia: An explorative study. J. Psychosom. Obs. Gynaecol..

[10] Brusse I., Duvekot J., Jongerling J., Steegers E., De Koning I. (2008). Impaired maternal cognitive functioning after pregnancies complicated by severe pre-eclampsia: A pilot case-control study. Acta Obstet. Et. Gynecol. Scand..

[11] Fields J.A., Garovic V.D., Mielke M.M., Kantarci K., Jayachandran M., White W.M., Butts A.M., Graff-Radford J., Lahr B.D., Bailey K.R. (2017). Preeclampsia and cognitive impairment later in life. Am. J. Obstet. Gynecol..

[12] Postma I.R., Bouma A., Ankersmit I.F., Zeeman G.G. (2014). Neurocognitive functioning following preeclampsia and eclampsia: A long-term follow-up study. Am. J. Obstet. Gynecol..

[13] Postma I.R., Groen H., Easterling T.R., Tsigas E.Z., Wilson M.L., Porcel J., Zeeman G.G. (2013). The brain study: Cognition, quality of life and social functioning following preeclampsia; An observational study. Pregnancy Hypertens..

[14] Postma I.R., Wessel I., Aarnoudse J.G., Zeeman G.G. (2010). Neurocognitive functioning in women with a history of eclampsia: Executive functioning and sustained attention. Am. J. Perinatol..

[15] Roes E.M., Raijmakers M.T., Schoonenberg M., Wanner N., Peters W.H., Steegers E.A. (2005). Physical well-being in women with a history of severe preeclampsia. J. Matern. Fetal Neonatal Med..

[16] Johnson A.C., Tremble S.M., Cipolla M.J. (2022). Experimental Preeclampsia Causes Long-Lasting Hippocampal Vascular Dysfunction and Memory Impairment. Front. Physiol..

[17] Huang C., Wei K., Lee P.M.Y., Qin G., Yu Y., Li J. (2022). Maternal hypertensive disorder of pregnancy and mortality in offspring from birth to young adulthood: National population based cohort study. BMJ.

[18] Korzeniewski S.J., Sutton E., Escudero C., Roberts J.M. (2022). The Global Pregnancy Collaboration (CoLab) symposium on short- and long-term outcomes in offspring whose mothers had preeclampsia: A scoping review of clinical evidence. Front. Med..

[19] Santillan M.K., Santillan D.A., Sigmund C.D., Hunter S.K. (2009). From molecules to medicine: A future cure for preeclampsia?. Drug. News Perspect..

[20] Kadyrov M., Kingdom J.C., Huppertz B. (2006). Divergent trophoblast invasion and apoptosis in placental bed spiral arteries from pregnancies complicated by maternal anemia and early-onset preeclampsia/intrauterine growth restriction. Am. J. Obstet. Gynecol..

[21] Darmochwal-Kolarz D., Kludka-Sternik M., Tabarkiewicz J., Kolarz B., Rolinski J., Leszczynska-Gorzelak B., Oleszczuk J. (2012). The predominance of Th17 lymphocytes and decreased number and function of Treg cells in preeclampsia. J. Reprod. Immunol..

[22] Wallace K., Richards S., Dhillon P., Weimer A., Edholm E.S., Bengten E., Wilson M., Martin J.N., LaMarca B. (2011). CD4+ T-helper cells stimulated in response to placental ischemia mediate hypertension during pregnancy. Hypertension.

[23] Laresgoiti-Servitje E., Gómez-López N., Olson D.M. (2010). An immunological insight into the origins of pre-eclampsia. Hum. Reprod. Update.

[24] Redman C.W., Sargent I.L. (2010). Immunology of pre-eclampsia. Am. J. Reprod. Immunol..

[25] Scroggins S.M., Santillan D.A., Lund J.M., Sandgren J.A., Krotz L.K., Hamilton W.S., Devor E.J., Davis H.A., Pierce G.L., Gibson-Corley K.N. (2018). Elevated vasopressin in pregnant mice induces T-helper subset alterations consistent with human preeclampsia. Clin. Sci..

[26] Coombes J.L., Powrie F. (2008). Dendritic cells in intestinal immune regulation. Nat. Rev. Immunol..

[27] Banchereau J., Briere F., Caux C., Davoust J., Lebecque S., Liu Y.J., Pulendran B., Palucka K. (2000). Immunobiology of dendritic cells. Annu. Rev. Immunol..

[28] Joffre O.P., Segura E., Savina A., Amigorena S. (2012). Cross-presentation by dendritic cells. Nat. Rev. Immunol..

[29] Morelli A.E., Thomson A.W. (2007). Tolerogenic dendritic cells and the quest for transplant tolerance. Nat. Rev. Immunol..

[30] Rutella S., Lemoli R.M. (2004). Regulatory T cells and tolerogenic dendritic cells: From basic biology to clinical applications. Immunol. Lett..

[31] Steinman R.M., Hawiger D., Nussenzweig M.C. (2003). Tolerogenic dendritic cells. Annu. Rev. Immunol..

[32] Stenger E.O., Turnquist H.R., Mapara M.Y., Thomson A.W. (2012). Dendritic cells and regulation of graft-versus-host disease and graft-versus-leukemia activity. Blood.

[33] Cools N., Ponsaerts P., Van Tendeloo V.F., Berneman Z.N. (2007). Balancing between immunity and tolerance: An interplay between dendritic cells, regulatory T cells, and effector T cells. J. Leukoc. Biol..

[34] Torres-Aguilar H., Aguilar-Ruiz S.R., Gonzalez-Perez G., Munguia R., Bajana S., Meraz-Rios M.A., Sanchez-Torres C. (2010). Tolerogenic dendritic cells generated with different immunosuppressive cytokines induce antigen-specific anergy and regulatory properties in memory CD4+ T Cells. J. Immunol..

[35] Liu S., Zhang S., Hong L., Diao L., Cai S., Yin T., Zeng Y. (2023). Characterization of progesterone-induced dendritic cells in metabolic and immunologic reprogramming. J. Reprod. Immunol..

[36] Marin E., Bouchet-Delbos L., Renoult O., Louvet C., Nerriere-Daguin V., Managh A.J., Even A., Giraud M., Vu Manh T.P., Aguesse A. (2019). Human Tolerogenic Dendritic Cells Regulate Immune Responses through Lactate Synthesis. Cell. Metab..

[37] Navarro-Barriuso J., Mansilla M.J., Quirant-Sanchez B., Teniente-Serra A., Ramo-Tello C., Martinez-Caceres E.M. (2020). Vitamin D3-Induced Tolerogenic Dendritic Cells Modulate the Transcriptomic Profile of T CD4(+) Cells towards a Functional Hyporesponsiveness. Front. Immunol..

[38] Fujita S., Seino K., Sato K., Sato Y., Eizumi K., Yamashita N., Taniguchi M. (2006). Regulatory dendritic cells act as regulators of acute lethal systemic inflammatory response. Blood.

[39] Fujita S., Yamashita N., Ishii Y., Sato Y., Sato K., Eizumi K., Fukaya T., Nozawa R., Takamoto Y., Taniguchi M. (2008). Regulatory dendritic cells protect against allergic airway inflammation in a murine asthmatic model. J. Allergy Clin. Immunol..

[40] Lan Y.Y., Wang Z., Raimondi G., Wu W., Colvin B.L., de Creus A., Thomson A.W. (2006). “Alternatively activated” dendritic cells preferentially secrete IL-10, expand Foxp3+CD4+ T cells, and induce long-term organ allograft survival in combination with CTLA4-Ig. J. Immunol..

[41] Chorny A., Gonzalez-Rey E., Fernandez-Martin A., Ganea D., Delgado M. (2006). Vasoactive intestinal peptide induces regulatory dendritic cells that prevent acute graft-versus-host disease while maintaining the graft-versus-tumor response. Blood.

[42] Sato K., Yamashita N., Baba M., Matsuyama T. (2003). Regulatory dendritic cells protect mice from murine acute graft-versus-host disease and leukemia relapse. Immunity.

[43] Scroggins S.M., Olivier A.K., Meyerholz D.K., Schlueter A.J. (2013). Characterization of regulatory dendritic cells that mitigate acute graft-versus-host disease in older mice following allogeneic bone marrow transplantation. PLoS ONE.

[44] Wan X., Bao L., Ma G., Long T., Li H., Zhang Y., Jiang H. (2022). Tolerogenic dendritic cells alleviate collagen-induced arthritis by forming microchimerism and affecting the expression of immune checkpoint molecules. Eur. J. Immunol..

[45] Chorny A., Gonzalez-Rey E., Fernandez-Martin A., Pozo D., Ganea D., Delgado M. (2005). Vasoactive intestinal peptide induces regulatory dendritic cells with therapeutic effects on autoimmune disorders. Proc. Natl. Acad. Sci. USA.

[46] Sandgren J.A., Deng G., Linggonegoro D.W., Scroggins S.M., Perschbacher K.J., Nair A.R., Nishimura T.E., Zhang S.Y., Agbor L.N., Wu J. (2018). Arginine vasopressin infusion is sufficient to model clinical features of preeclampsia in mice. JCI Insight.

[47] Santillan M.K., Santillan D.A., Scroggins S.M., Min J.Y., Sandgren J.A., Pearson N.A., Leslie K.K., Hunter S.K., Zamba G.K., Gibson-Corley K.N. (2014). Vasopressin in preeclampsia: A novel very early human pregnancy biomarker and clinically relevant mouse model. Hypertension.

[48] Ramdin S., Naicker T., Pillay V., Singh S.D., Baijnath S., Mkhwanazi B.N., Govender N. (2022). Physiological characterization of an arginine vasopressin rat model of preeclampsia. Syst. Biol. Reprod. Med..

[49] Zhang Y., Liu Z., Tian M., Hu X., Wang L., Ji J., Liao A. (2018). The altered PD-1/PD-L1 pathway delivers the ‘one-two punch’ effects to promote the Treg/Th17 imbalance in pre-eclampsia. Cell. Mol. Immunol..

[50] Zhang X., Li M., Lian D., Zheng X., Zhang Z.X., Ichim T.E., Xia X., Huang X., Vladau C., Suzuki M. (2008). Generation of therapeutic dendritic cells and regulatory T cells for preventing allogeneic cardiac graft rejection. Clin. Immunol..

[51] Littlejohn N.K., Siel R.B., Ketsawatsomkron P., Pelham C.J., Pearson N.A., Hilzendeger A.M., Buehrer B.A., Weidemann B.J., Li H., Davis D.R. (2013). Hypertension in mice with transgenic activation of the brain renin-angiotensin system is vasopressin dependent. Am. J. Physiol. Regul. Integr. Comp. Physiol..

[52] Santillan M.K., Pelham C.J., Ketsawatsomkron P., Santillan D.A., Davis D.R., Devor E.J., Gibson-Corley K.N., Scroggins S.M., Grobe J.L., Yang B. (2015). Pregnant mice lacking indoleamine 2,3-dioxygenase exhibit preeclampsia phenotypes. Physiol. Rep..

